# Social capital and willingness to participate in COVID-19 vaccine trials: an Italian case-control study

**DOI:** 10.1186/s12889-022-14562-2

**Published:** 2022-11-21

**Authors:** Marco Terraneo, Valeria Quaglia, Giampaolo Nuvolati, Marco Bani, Selena Russo, Maria Grazia Strepparava, Serena Capici, Rebecca Cavaliere, Marina Elena Cazzaniga

**Affiliations:** 1 Department of Sociology and Social Research, University of Milano-Bicocca, Milan, Italy; 2 Department of Political Science, Communication and International Relations, University of Macerata, Macerata, Italy; 3 School of Medicine and Surgery, University of Milano-Bicocca, Milan, Italy; 4 Clinical Psychology Unit, ASST Monza, University of Milano-Bicocca, Monza, Italy; 5 Phase 1 Research Centre, ASST Monza, via Cadore 48, 20900 Monza, Italy; 6 Royal College of Surgeons, University of Medicine and Health Sciences, Dublin, Ireland

**Keywords:** COVID-19, Vaccine, Vaccine trials willingness, Social capital, Italy

## Abstract

**Background:**

What leads healthy people to enter in a volunteer register for clinical trials? This study aimed to investigate the relationship between the decision to volunteer in clinical trials for a COVID-19 vaccine and social capital, in a sample of healthy volunteers in Italy. Since social capital is characterized by trust, reciprocity, and social and political participation, we claim that it is key in leading individuals to actively take action to protect public health, and to take a risk for the (potential) benefit not only of themselves but for the entire community.

**Methods:**

This study was conducted through the administration of a questionnaire to healthy volunteers registered for a phase 1 clinical trial for a COVID-19 vaccine in the Unit Research Centre of ASST-Monza, in September 2020. The primary purpose of a phase 1 study is to evaluate the safety of a new drug candidate before it proceeds to further clinical studies. To approximate a case–control study, we randomly matched the 318 respondents to healthy volunteers (cases) with 318 people randomly selected by Round 9 of the European Social Survey (controls), using three variables, which we considered to be associated with the decision to volunteer: gender, age, and education level. To execute this matching procedure, we used the “ccmatch” module in STATA.

**Results:**

The findings highlight the positive impact of social capital in the choice of healthy individuals to volunteer in COVID-19 vaccine clinical trials. Controlling for possible confounding factors, some exemplary results show that people with a high level of general trust have a greater likelihood of volunteering compared to people with low trust (OR = 2.75, CI = 1.58–4.77); we also found that it is more probable that volunteers are people who have actively taken action to improve things compared with people who have not (for individuals who did three or more actions: OR = 7.54, CI = 4.10–13.86). People who reported voting (OR = 3.91, CI = 1.70–8.99) and participating in social activities more than other people of their age (OR = 2.89, CI = 1.82–4.60) showed a higher probability to volunteer.

**Conclusions:**

Together with the adoption of urgent health measures in response to COVID-19, government policymakers should also promote social capital initiatives to encourage individuals to actively engage in actions aimed at protecting collective health. Our findings make an empirical contribution to the research on vaccines and its intersection with social behaviour, and they provide useful insights for policymakers to manage current and future disease outbreaks and to enhance the enrolment in vaccine trials.

## Background

Coronavirus disease 2019 (COVID-19) is a disease caused by a new coronavirus, severe acute respiratory syndrome coronavirus 2 (SARS-CoV-2). It was discovered after an outbreak in Wuhan, China, in December 2019. SARS-CoV-2 is part of a larger family of coronaviruses that cause illnesses ranging from the common cold to more severe infections in humans. As of 17 July 2022, over 559 million confirmed cases and over 6.3 million deaths have been reported globally (https://www.who.int/publications/m/item/weekly-epidemiological-update-on-covid-19%2D%2D-20-july-2022, last viewed 29/07/2022). The COVID-19 pandemic represents a global challenge and an unprecedented global health crisis that has caused major worldwide disruption. Beginning in early 2020, governments introduced a series of measures to reduce the number of infections, including physical distancing, limits to freedom of movement locally and internationally, and the closure of social spaces (such as schools and restaurants). In addition to preventive measures, vaccination is the main strategy adopted to provide protection from the virus. In times of crisis like the current one, the participation of healthy populations in clinical trials is crucial for the development of new vaccines that will help to end the pandemic. Since vaccine trials rely on volunteers, understanding the reasons that influence their willingness to participate is fundamental for informing scientific decisions about COVID-19 vaccine trials as well as for identifying possible social factors associated with future vaccine uptake [1, 2].

Past studies on individual decision making regarding medical and vaccine trials show that, typically, financial motivation is prevalent [3]. But economic reward is not the only reason that influences the willingness of healthy volunteers to participate in clinical trials: other motivations include, for example, the desire to contribute to science and medicine; the desire to participate in something important; curiosity or the desire to learn more about science and medicine [3]; access to health care, social support, and trust [4]; the desire to help others (altruism) [5–8]; and personal health benefits [8–10].

Compared to other trials, participation in COVID-19 vaccine trials presents some specific issues: on the one hand, individuals who suffer from some form of disease might be motivated to participate mainly to obtain benefits for their own health [11], and on the other hand, COVID-19 vaccine trials might be different because while potential participants are healthy, they are willing to put their health at risk to the (potential) benefit of not only themselves but also that of the community.

Recent studies that have focused on individuals’ motivations to enrol in COVID-19 vaccine trials found that individuals may volunteer for different reasons, including for instance a desire to actively contribute to ending the pandemic and returning to pre-pandemic “normalcy”; an expression of dimensions of identity such as being a helper, supporting medicine/vaccines development, and trusting science and in its institutions [12, 13]; a perception of being at risk of COVID-19 infection; COVID-19 prosocial behaviours [1]; the hope of being protected against COVID-19; altruism; and the opportunity to get health care [14].

In our study, we hypothesize that social capital could play a significant role in the decision of healthy individuals to participate in COVID-19 vaccine trials. Despite the fact that social capital has been defined in different ways over recent decades, overall there is unanimous agreement in recognizing the value of social networks and the associated norms of reciprocity. The most commonly used definition of social capital is one provided by Putnam ([15]: p. 6) that refers to “features of social organization such as networks, norms and trust, that facilitate coordination for mutual benefit”.

Social capital creates value for the individuals who are part of social networks, but also for others. Scholars have demonstrated the relevance of social capital in different domains, such as economic and social outcomes, but as Putnam ([16], p.326) states, “In none is the importance of social connectedness so well established as in the case of health and well-being”. Higher levels of social capital in fact seem to produce healthier societies [17]. When the physical capital of a population is eroded or put at risk by a health crisis, social capital becomes relevant in increasing individuals’ concern for others and in engaging in practices that aim to improve the overall situation.

Previous international studies have demonstrated the relevance of social capital in handling past outbreaks [18, 19] as well as the current COVID-19 pandemic [20–24]. For example, in line with other studies [25], Makridis and Wu [26] investigated the impact of social capital on the growth rate of COVID-19 infections and found that, instead of leading to an increased spread of the virus because of a higher number of social interactions, social capital had an important negative effect on the number of infections and viral spread, because individuals were more willing to engage in preventive measures against COVID-19 such as hygienic practices and physical distancing. In turn, Wu [24] found that, in the U.S., states with higher levels of both social capital and social trust showed a more desirable response to COVID-19, and more specifically they tended to have higher testing rates.

Other studies have demonstrated that higher levels of social capital were positively associated with willingness to get vaccinated against COVID-19 [27] and with willingness to undergo booster shots [28], under the assumption that such vaccination would be important to protect not only the health of those who receive it but also that of their families, friends, and the community as a whole.

Recognizing the importance of social capital in developing effective strategies to combat COVID-19 implies acknowledging the limitations of top-down policies and the need to complement the public health responses with measures that take into account the relevance of trust, social participation, and cohesion. Since the willingness to participate in vaccine clinical trial implies not only (potential) personal health benefits but also a form of self-sacrifice for the good of all, in this article we argue that social capital is positively associated with the participation of healthy people in COVID-19 vaccine clinical trials in Italy.

The aim of this study is to describe the relationship between type and level of social capital and the decision to volunteer in a clinical trial for a COVID-19 vaccine, controlling for a set of possible confounders. Specifically, we are interested in: a) evaluating whether people with higher levels of social capital are more likely to volunteer than individuals with low social capital; and b) establishing whether this propensity varies according to the type of social capital considered. As described in detail below, to assess the effect of social capital we control for some individual socio-demographic characteristics.

## Methods

Between June and September 2020, an open call was launched for healthy volunteers for a phase 1 clinical trial of a COVID-19 vaccine by the Unit Research Centre of ASST-Monza (Azienda Socio-sanitaria Territoriale - Territorial Social Healthcare Company).

The primary purpose of a phase 1 study is to evaluate the safety of a new drug candidate before it proceeds to further clinical studies. Our Unit Research Centre was selected to participate in a first-in-human phase 1 study on a DNA anti-COVID-19 vaccine. However, the subjects were part of a healthy volunteer registry that could have been used for any study that required the participation of healthy volunteers.

People were visited by a medical team to establish that their physical health and psychological conditions were suitable for inclusion in the registry of healthy volunteers. Health status was assessed by collecting their medical history, information about concomitant medications, and, if available, blood tests from a clinical visit. Subjects with a personal history of previous malignancies, uncontrolled diabetes, hypertension, or cardiac disease were excluded, as were those with mental disorders.

Another eligibility criterion for entry in the healthy volunteer registry was being able to provide informed consent. At the end of the medical interview, all subjects were informed about the possibility to be enrolled, after a second and more extensive clinical and instrumental evaluation, in the phase 1 DNA vaccine study. Medical staff informed them about the procedure of electroporation and its potential side effects in terms of pain and bruising at the injection site. They were also informed about the preliminary results obtained in animal models of the immunogenicity achieved with the DNA vaccine. The study was a first-in-human phase 1 trial; therefore, at the moment of enrolment, no other information was available.

Only subjects who fulfilled the criteria for study enrolment and who were fully eligible received a fee for their participation. For the recruitment process itself, no fee was offered. Individuals who did not meet these criteria were excluded from the registry.

A self-administered online questionnaire was sent to 478 people included in the healthy volunteers registry. There was only one criterion for exclusion from the survey: noncompletion of the whole interview. In total, 320 complete interviews were collected, for an overall response rate of 66,9%.

The research group designed a computer-assisted web interviewing (CAWI) as a questionnaire using Google Forms. The questionnaire, which was estimated to take 20 minutes for completion, was anonymous and, as part of a wider cross-sectional study on sociological and psychological predictors, was composed of eight sections, focusing on 1) risk perception, fear, and perceived severity of COVID-19; 2) motivation to participate in a clinical trial; 3) trust in investigators, doctors, institutions, and pharmaceutical companies; 4) social capital; 5) religiosity; 6) attachment style; 7) health literacy, health locus of control; and 8) the participant’s socio-demographic information. These questionnaire sections covered a wide range of issues related to trial participation, as discussed in scientific literature on this topic. Specifically, we included in the questionnaire three key dimensions from a sociological perspective: risk perception, social capital, and religiosity.

E-mail invitations were sent to every healthy volunteer in the registry and included a link to the questionnaire, use of which thereby acknowledged their agreement to participate in the survey. A follow-up e-mail reminder was sent during the data collection to encourage participation. The questionnaire was available online throughout the month of June 2021.

As we had a homogeneous sample, i.e. all people were healthy volunteers willing to participate in a COVID-19 vaccine trial, in accordance with our research aims we approximated a case–control study. Our analytical strategy was the following.

We analysed several datasets that reported data for Italy in which there were questions concerning social capital like those present in our study. We opted for Round 9 of the European Social Survey (ESS), whose data were collected in 2018–2019, in which six questions overlapped with questions from our volunteers’ survey.

The six social capital variables considered are these:

- General trust (“Generally speaking, would you say that most people can be trusted, or that you can’t be too careful in dealing with people?”). The original score ranges from 0 to 10, where 0 refers to “you can’t be too careful” and 10 corresponds to “most people can be trusted”. The variable was coded by three levels of trust: low (score 0–3), medium (4–6), and high (7–10).
- Reciprocity-1 (“Do you think that most people would try to take advantage of you if they got the chance, or would they try to be fair?”). The original score ranges from 0 to 10, where 0 means “most people would take advantage of you” and 10 means that most people would try to be fair. The variable was coded by three levels of reciprocity: low (score 0–3), medium (4–6), and high (7–10).
- Reciprocity-2 (“Would you say that most of the time people try to be helpful or that they are mostly looking out for themselves?”). The original score ranges from 0 to 10, where 0 means that people mostly look out for themselves and 10 that people mostly try to be helpful. The variable was coded by three levels: low (0–3), medium (4–6), and high (7–10).
- Improve things (“There are different ways of trying to improve things in Italy or help prevent things from going wrong. During the last 12 months, have you done any of the following? –contacted a politician, government or local government official? –worked in a political party or action group? –worked in another organisation or association? –worn or displayed a campaign badge/sticker? –signed a petition? –taken part in a lawful public demonstration? –boycotted certain products?”). We constructed an additive measure summing the original answers (0 things not done, 1 thing done), and the total score, which varied between 0 and 7, was coded by four levels: 0 things done, 1 thing done, 2 things done, 3–7 things done.
- Vote (“Did you vote in the last Italy national election?”). The variable was coded as 1, “voted”, and 0, “did not vote”. Social activities (“Compared to other people of your age, how often would you say you take part in social activities?”). The original score varies between 1, “much less than most”, to 5, “much more than most”. The variable was coded by two levels: less than or about the same as other people (score 1–3), and more than other people (4–5).

Moreover, we constructed an additive index of social capital (SCI), summing the normalized scores of the six variables previously described according to a formula that takes into account the different number of modalities of the summed variables— X_i_ − X_min_/X_max_ − X_min_— and then calculating the tertiles of overall score. Therefore, in the first tertile we found people with a low level of social capital, in the second one those with a medium level of social capital, in the third tertile people with a high level of social capital.

There were 2626 total cases available in the ESS sample for Italy. Next, we selected individuals with the same age interval of volunteers (19–73 years), reducing the sample to 2491 individuals. The last step was to randomly match cases (data from volunteer survey) and controls (data from selected ESS survey) using three variables, which we considered to be associated with a decision to volunteer, as criteria on which to match cases and controls: gender, age (in decile), and education level (in three classes: middle school or less, high school, bachelor’s degree or more). To execute this matching procedure, we used the “*ccmatch*” module in STATA. This procedure is used to randomly match cases and controls based on specified criteria. Specifically, in this work, we randomly matched cases and controls based on gender, age, and education level; therefore these three variables were used as a criterion to match cases and controls [29]. Of the initial 320 cases, 318 were matched; therefore our final sample included 636 individuals, 318 cases and 318 controls. In Table 1 (related to matching variables) we report summary statistics for the variables of interest, distinguishing between cases and controls.

**Table 1:** Descriptive statistics of controls and cases by social capital variables

	Controls					Cases				
	Mean	SD	Min	Max	N	Mean	SD	Min	Max	N
General trust	2,01	0,71	1	3	317	2,25	0,70	1	3	318
Reciprocity - 1	2,00	0,69	1	3	317	2,41	0,64	1	3	318
Reciprocity - 2	1,82	0,69	1	3	317	2,14	0,73	1	3	318
Improve things	0,72	1,04	0	3	310	1,54	1,18	0	3	294
Vote	0,84	0,36	0	1	287	0,95	0,21	0	1	307
Social activities	1,12	0,33	1	2	316	1,31	0,46	1	2	303
Social capital index	1,64	0,80	1	3	280	2,22	0,79	1	3	273

To test our hypothesis, we tabulated the odds of failure (odds ratios, ORs) against a categorical explanatory variable, and 95% confidence intervals (CIs) were calculated. In this context, failure was to be a healthy vaccine volunteer, and the explanatory variables were the different social capital dimensions. This means that we estimated the risk of people becoming volunteers for a COVID-19 vaccine clinical trial according to their type and level of social capital. We also conducted an approximate chi-squared test of homogeneity of odds and a test for linear trend of the log odds against the numerical code used for the categories, whose differences were considered significant at *p*-value < 0.05. Moreover, adjusted Mantel–Haenszel odds ratios for three variables—employment status (distinguishing between employed, unemployed, and inactivity), risk perception (high, medium, and low), and religiosity (belonging or not belonging to a specific religion)—along with a (score) test for trend were calculated. Descriptive statistics of control variables are reported in Table 2 (related to matching variables).

**Table 2:** Descriptive statistics of controls and cases by matching and confounding variables

		Controls					Cases				
		Mean	SD	Min	Max	N	Mean	SD	Min	Max	N
Matching variables	Age (in deciles)	5,51	2,47	1	10	318	5,51	2,47	1	10	318
	Gender	1,38	0,49	1	2	318	1,38	0,49	1	2	318
	Education (in classes)	2,37	0,62	1	3	318	2,37	0,62	1	3	318
Confounding variables	Employment status	1,65	1,16	1	4	317	1,53	1,09	1	4	315
	Risk propensity	1,82	0,63	1	3	310	2,17	0,69	1	3	318
	Religiosity	1,28	0,45	1	2	314	1,44	0,50	1	2	256

Statistical analysis was performed with STATA 17.0 (StataCorp LP, College Station, TX, USA).

## Results

**Fig. 1:**
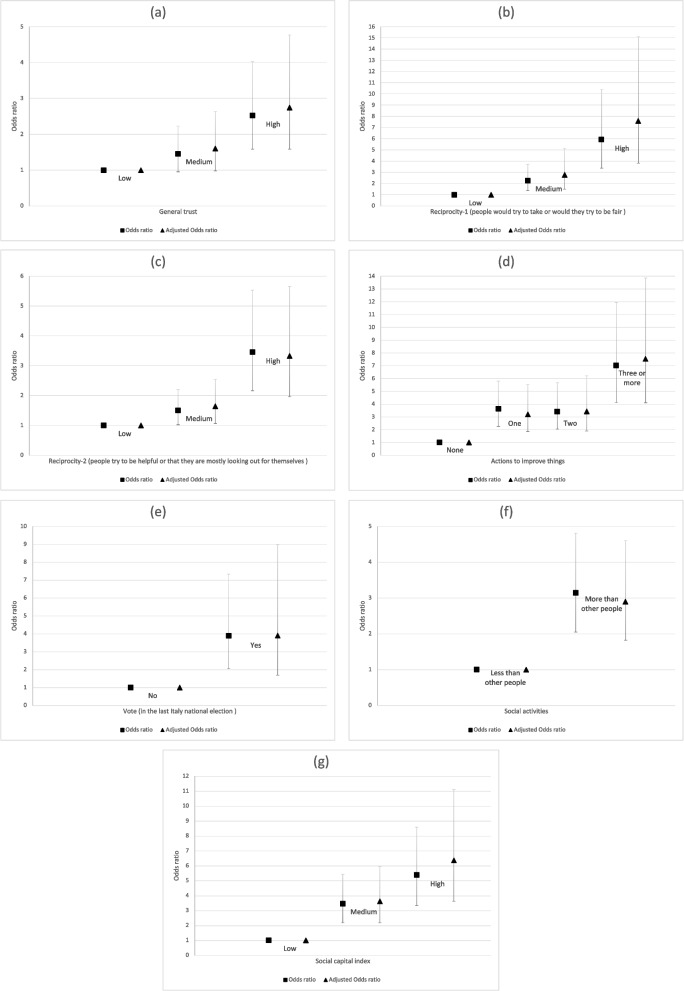
ORs and 95% CIs of estimated likelihood of participating in a COVID-19 vaccine clinical trial by social capital. Panel (**a**) General trust; Panel (**b**) Reciprocity – 1; Panel (**c**) Reciprocity – 2; Panel (**d**) Improve things; Panel (**e**) Vote; Panel (**f**) Social activities; Panel (**g**) Social capital index

Figure 1 shows the findings of our analysis. It displays ORs and 95% CIs of the estimated likelihood of participating in a COVID-19 vaccine clinical trial according to the level of individual social capital. Specifically, squares represent unadjusted ORs and triangles represent adjusted ORs for employment status, risk perception, and religiosity. It is fundamental to highlight that by construction, cases and controls were merged for gender, age, and level education; therefore cases and controls in all analyses are adjusted for these three variables.

In Fig. 1, panel (a), we can see that people with a medium level (OR = 1.46, CI = 0.95–2.23, OR = 1.60, CI = 0.98–2.63, though at the limit of statistical significance, *p* = 0.08 and *p* = 0.06 for raw and adjusted estimates, respectively) and a high level of general trust (OR = 2.53, CI = 1.59–4.02, OR = 2.75, CI = 1.58–4.77) show a greater likelihood of volunteering than people with low trust.

The same pattern was found also for Reciprocity-1 and Reciprocity-2 social capital variables; see Fig. 1, panels (b) and (c). For Reciprocity-1, the estimated ORs are the following for raw estimates: OR = 2.26, CI = 1.36–3.73 for medium level and OR = 5.93, CI = 3.38–10.40 for high level; for adjusted estimates: OR = 2.78, CI = 1.50–5.15 for medium level and OR = 7.60, CI = 3.82–15.12 for high level). For Reciprocity-2, we find these values: OR = 1.50, CI = 1.02–2.20 for medium level and OR = 1.64, CI = 1.06–2.54 for high level; for adjusted estimates: OR = 3.45, CI = 2.16–5.53 for medium level and OR = 3.35, CI = 1.97–5.66 for high level. The association between social capital and propensity to volunteer is confirmed by looking at actions people reported taking to improve things (Fig. 1, panel (d)). It is more probable that volunteers are people who took actions to make things better compared with people who did not. This probability increased significantly for individuals who took three or more actions (OR = 7.02, CI = 4.12–7.96 for raw estimates and OR = 7.54, CI = 4.10–13.86 for adjusted estimates). People who voted (OR = 3.89, CI = 2.06–7.34 for raw estimates and OR = 3.91, CI = 1.70–8.99 for adjusted estimates) and participated in social activities more than other people of their age (OR = 3.15, CI = 2.05–4.81 for raw estimates and OR = 2.89, CI = 1.82–4.60 for adjusted estimates) showed a higher probability to volunteer (Fig. 1, panels (e) and (f) respectively). Finally, looking at the social capital index represented in Fig. 1, panel (g), as we could expect, the higher the individual social capital, the greater the probability of volunteering. Also in this case, no difference emerged when comparing the model unadjusted for confounding variables (for medium social capital index: OR = 3.45, CI = 2.19–5,43, for high social capital index: OR = 5.38, CI = 3.36–8.60) and the model controlled for employment status, risk propensity, and religiosity (for medium social capital index: OR = 3.62, CI = 2.19–5.96, for high social capital index: OR = 6.36, CI = 3.64–11.12).

In Table 3 we display for each model an approximate chi-squared test of homogeneity of odds (only for the unadjusted model) and a test for linear trend of the log odds. Both tests achieved statistical significance (*p* < 0.05) for all models analysed in this work. The test of homogeneity clearly indicates that the odds of participating as volunteers in COVID-19 vaccine trials differ by level of social capital (specific for each social capital variable), and the test for trend, both for unadjusted and adjusted models, indicates a significant increase in odds with increasing social capital (also in this case specific for social capital variable considered in the analysis). Therefore, these tests suggest a high “dose–response” relation between the social capital and the decision to volunteer for vaccine clinical trials.

**Table 3:** Test of homogeneity of odds (only for unadjusted model) and a test for linear trend of the log odds by social capital variables

	Unadjusted model				Adjusted model	
	Test of homogeneity (equal odds)		Score test for trend of odds		Score test for trend of odds	
	chi2	Pr > chi2	chi2(1)	Pr > chi2	chi2	Pr > chi2
General trust	17.93	0.0001	17.64	0.0000	15.34	0.0001
Reciprocity - 1	56.43	0.0000	56.06	0.0000	55.51	0.0000
Reciprocity - 2	31.69	0.0000	30.07	0.0000	22.15	0.0000
Improve things	82.41	0.0000	72.80	0.0000	60.91	0.0000
Vote	20.47	0.0000	20.47	0.0000	11.96	0.0005
Social activities	31.00	0.0000	31.00	0.0000	22.27	0.0000
Social capital index	67.71	0.0000	63.65	0.0000	50.98	0.0000

Summarizing, the results clearly reveal that social capital, in its different forms, is strongly associated with individuals’ decision to volunteer for vaccine clinical trials. Moreover, they also suggest that the effect persists when controlling for possible confounding factors, both for education, age, and gender—variables used to match cases and controls in this study—and for employment status, religiosity, and risk propensity, which we supposed could be associated with the choice to volunteer.

## Discussion

Our study aimed to determine the relationship between social capital and willingness to participate as volunteers in COVID-19 vaccine trials, under the assumption that vaccine clinical studies are crucial in overcoming the current as well as possible future pandemics. Our analysis of data from an Italian survey on healthy volunteers registered for a COVID-19 vaccine clinical trial (June–September 2020) shows that all the social capital variables considered—general trust, reciprocity, the will to improve things, political participation, and participation in social activities—significantly affect the willingness of healthy individuals to enrol in COVID-19 vaccine trials. In particular, we found that individuals with higher levels of social capital are more likely to be willing to volunteer in vaccine clinical trials compared to individuals with lower levels. More specifically, individuals with medium and high levels of general trust are more likely to volunteer than people with low trust. This result is in line with previous studies: Makridis and Wu [27] found that when individuals are embedded in social networks characterized by higher levels of faith in each other, they are more willing to show a greater concern for others and to adopt prevention and control measures. Similarly, in a pre-pandemic study, Aldric [30] found that in post-crisis times people with higher levels of social capital—in the form of trust—are more likely to share information with each other and to help the community. Social ties that enable trust are also crucial during a time of pandemic: trust is fundamental for public compliance on government measures and strategies to overcome the health emergency, including vaccine and drug trials.

The same pattern was found also for the two variables regarding reciprocity. In fact, reciprocity is a cornerstone of social capital: individuals who are connected to each other by networks of reciprocity are more likely to comply with the law because they assume that others would do the same. In the case under study, this result refers to the higher probability that an individual with a higher level of social capital—in the form of reciprocity—would be willing to risk his or her own health and take part in an anti-disease measure such as a COVID-19 vaccine trial with the expectation that others would do the same if necessary.

The remaining variables—the will to improve things, civic and political participation—all point in the same direction: individuals who more actively participate in solving social issues and improving collective well-being are also more willing to participate as volunteers in vaccine trials, as they could be more prone to engage in actions to support the development of science and to preserve public health.

These results are especially relevant considering the actual emergency responses that have been adopted by most governments, which have instead mainly focused on individual responsibility and on top-down measures to partially limit individuals’ freedom of movement to limit the spread of the virus. Our findings point to the need to integrate social capital into any policy measure aimed at enhancing adherence in vaccine trials.

Several methodological limitations should be considered when interpreting the results of this study. First, we selected the control cases sample using a statistical procedure based on three observed variables—gender, age, and level of education—but, although this strategy allowed us to compare the group of healthy volunteers with a group from the general population, it did not completely solve the problem of unobserved heterogeneity (i.e. selection bias). A second limitation refers to the fact that in a case–control study, controls should form a random sample from the population, for example, those at risk of developing the disease. In our work, the control group derived from a general population in which the probability for an individual to enter in a volunteer register for clinical trials could be different from that of the population to which healthy volunteers belong. If the probability of being a volunteer (the exposure status) is differentially distributed between cases and controls, this leads to a distortion of the exposure–outcome association. Third, the overall response rate of people in the phase 1 clinical trials healthy volunteers survey was 66,9% (320/478). If people who did not answer the questionnaire were systematically different (for observed and unobserved characteristics) from those who answered, then our estimates would be biased (i.e. attrition bias). Fourth, the relationship between social capital and the individual decision-making process regarding participation in phase 1 COVID-19 vaccine trials concerns only the variables present simultaneously in both datasets (i.e. the volunteer survey and the ESS surveys). In the questionnaire administered to the volunteers, the social capital was measured through a high number of questions in order to grasp the different types of social capital, but in this work, as there are only six common questions in the two questionnaires, an assessment of impact for all different types of social capital is not feasible.

Because of these limitations, the generalizability of results of this study should be carefully considered. In fact, as stated by Hulley and colleagues [31], for population-based case–control studies, the external validity (generalizability) is directly related to the underlying source population for cases and controls, and is related to the nature of the sampling frame from the source population, participation rates, and the characteristics of the non-participants.

However, the study provides the first evidence to date for a positive association between social capital and willingness to participate in COVID-19 vaccine trials in Italy. A better understanding of how social resources might mediate health outcomes during emergencies is important to advance science and medicine and to find solutions to protect the health of the population against current and future health emergencies.

## Conclusion

Our study showed an association between social capital and willingness to participate as volunteers in COVID-19 vaccine trials. These findings are particularly relevant as they may be useful to promote future participation of healthy individuals in vaccine trials, to advance medical knowledge and to find solutions to protect the health of the population against current and future health emergencies.

Our findings also have policy implications: in addition to taking immediate measures to prevent the spread of pandemics like COVID-19—such as physical distancing and the use of masks—policy makers should also consider the impact of social capital on promoting the engagement of individuals in actions aimed at collective well-being. In fact, limiting and ending a pandemic depends on several factors, among which are individuals’ behaviours and attitudes. It has been shown that preventive measures alone are not enough, and past research has found that social capital has a mediating role in determining individuals’ compliance with such measures [32]. Social capital should thus be urgently increased through policy actions: governmental decision makers need to consider that building trust, fostering mutual support, and promoting the political and social participation of citizens is key not only for participating in clinical trials but also for handling outbreaks, implementing emergency preparedness programmes, strengthening the social fabric, and increasing community cohesion and resilience.

## Data Availability

The dataset used and/or analysed during the current study available from the corresponding author on reasonable request.

## References

[1] Sun S, Lin D, Operario D (2021). Interest in COVID-19 vaccine trials participation among young adults in China: willingness, reasons for hesitancy, and demographic and psychosocial determinants. Prev Med Rep.

[2] Fadda M, Albanese E, Suggs LS (2020). When a COVID-19 vaccine is ready, will we all be ready for it?. Int J Public Health.

[3] Stunkel L, Grady C (2011). More than the money: a review of the literature examining healthy volunteer motivations. Contemp Clin Trials.

[4] Browne J, Rees CO, van Delden JJM, Agyepong I, Grobbee DE, Edwin A, Klipstein-Grobusch K, van der Graaf R (2019). The willingness to participate in biomedical research involving human beings in low-and middle-income countries: a systematic review. Tropical Med Int Health.

[5] Luchtenberg M, Maeckelberghe E, Locock L, Powell L, Eduard Verhage AA. Why young people participate in clinical trials: Els Maeckelberghe. Eur J Pub Health. 2016;26:25.

[6] Harro CD, Judson FN, Gorse GJ, Mayer KH, Kostman JR, Brown SJ, Koblin B, Marmor M, Bartholow BN, Popovic V (2004). Recruitment and baseline epidemiologic profile of participants in the first phase 3 HIV vaccine efficacy trial. J Acquir Immune Defic Syndr.

[7] Jenkins V, Fallowfield L (2000). Reasons for accepting or declining to participate in randomized clinical trials for cancer therapy. Br J Cancer.

[8] Bevan EG, Chee LC, McGhee SM, McInnes GT (1993). Patients’ attitudes to participation in clinical trials. Br J Clin Pharmacol.

[9] Locock L, Smith L (2011). Personal benefit, or benefiting others? Deciding whether to take part in clinical trials. Clin Trials.

[10] Strauss RP, Sengupta S, Kegeles S, McLellan E, Metzger D, Eyre S, Khanani F, Emrick CB, MacQueen KM (2001). Willingness to volunteer in future preventive HIV vaccine trials: issues and perspectives from three U.S. communities. J Acquir Immune Defic Syndr.

[11] Agrawal M, Grady C, Fairclough DL, Meropol NJ, Maynard K, Emanuel EJ (2006). Patients' decision-making process regarding participation in phase I oncology research. J Clin Oncol.

[12] Wentzell E, Racila AM (2021). The social experience of participation in a COVID-19 vaccine trial: subjects’ motivations, others’ concerns, and insights for vaccine promotion. Vaccine.

[13] Abu-Farha RK, Alzoubi KH, Khabour OF (2020). Public willingness to participate in COVID-19 vaccine clinical trials: a study from Jordan. Patient Prefer Adherence.

[14] Kitonsa J, Kamacooko O, Bahemuka UM, Kibengo F, Kakande A, Wajja A (2021). Willingness to participate in COVID-19 vaccine trials: a survey among a population of healthcare workers in Uganda. PLoS One.

[15] Putnam RD (1994). Social capital and public affairs. Bull Am Acad Arts Sci.

[16] Putnam RD (2000). Bowling alone: the collapse and revival of American community.

[17] Kawachi I, Subramanian SV, Kim D (2008). Social capital and health.

[18] Vinck P, Pham PN, Bindu KK, Bedford J, Nilles EJ (2019). Institutional trust and misinformation in the response to the 2018-19 Ebola outbreak in north Kivu, DR Congo: a population-based survey. Lancet Infect Dis.

[19] Blair R, Benjamin S, Morse LL, Tsai L (2016). Public health and public trust: survey evidence from the Ebola virus disease epidemic in Liberia. Soc Sci Med.

[20] Fraser T, Aldrich DP, Page-Tan C (2021). Bowling alone or distancing together? The role of social capital in excess death rates from COVID19. Soc Sci Med.

[21] Bai JJ, Jin W, Wan C (2020). The impact of social capital on individual responses to COVID-19 pandemic: Evidence from social distancing.

[22] Varshney LR, Socher R. COVID-19 growth rate decreases with social capital. medRxiv. 2020. 10.1101/2020.04.23.20077321.

[23] Wu C, Wilkes R, Fairbrother M, Giordano G. Social capital, trust, and state coronavirus testing. Contexts. Accessed on May 12, 2022: https://contexts.org/blog/healthcare-and-critical-infrastructure/#wu.

[24] Wu C (2021). Social capital and COVID-19: a multidimensional and multilevel approach. Chin Sociol Rev.

[25] Wong ASY, Kohler JC (2020). Social capital and public health: responding to the COVID-19 pandemic. Glob Health.

[26] Makridis CA, Wu C (2021). How social capital helps communities weather the COVID-19 pandemic. PLoS One.

[27] Aguilar Ticona JP, Nery N, Victoriano R, Fofana MO, Ribeiro GS, Giorgi E, Reis MG, Ko AI, Costa F (2021). Willingness to get the COVID-19 vaccine among residents of slum settlements. Vaccines.

[28] Hu T, Chuanxue L, Yang Z, Chow C, Zhipenf L, You C (2022). An analysis of the willingness to the COVID-19 vaccine booster shots among urban employees: evidence from a megacity H in eastern China. Int J Environ Res Public Health.

[29] Cook, DE. CCMATCH: Stata module to match cases and controls using specified variables. Statistical Software Components S457372, Boston College Department of Economics, 2011. revised 27 Jan 2015.

[30] Aldrich DP (2010). Fixing recovery: social capital in post-crisis resilience. J Homel Secur.

[31] Hulley SB, Cummings SR, Browner WS, Grady DG, Newman TB (2007). Designing clinical research.

[32] Bernados JR, S., Ocampo, L. (2022). How do people decide on getting vaccinated? Evaluating the COVID-19 vaccination program through the lens of social capital theory. Soc Sci.

